# Enhanced Expression of *CNTD2/CCNP* Predicts Poor Prognosis in Bladder Cancer Based on the GSE13507

**DOI:** 10.3389/fgene.2021.579900

**Published:** 2021-02-03

**Authors:** Mancheng Gong, Erlin Song, Guiying Huang, Wenjun Ni, Wenjing Dong, Runqiang Yuan

**Affiliations:** ^1^Department of Urology, The People’s Hospital of Zhongshan, Zhongshan, China; ^2^Department of Urology, First Affiliated Hospital of Harbin Medical University, Harbin, China; ^3^Department of Urology, The People’s Hospital of Zhuhai, Zhuhai, China; ^4^Department of Oncology, The People’s Hospital of Zhongshan, Zhongshan, China

**Keywords:** *CNTD2*, bladder cancer, bioinformatics analysis, prognosis, survival

## Abstract

Bladder cancer is one of the most common urogenital malignancies in the world, and there are no adequate prognostic indicators. *CNTD2* is one of the atypical cyclins, which may be related to the cell cycle and even the development of cancers. Early studies have shown that *CNTD2* is closely related to the occurrence and development of many malignant tumors. However, the mechanism of *CNTD2* in bladder cancer has not been reported. In our research, we explored the different expressions of *CNTD2* between 411 bladder cancers and 19 normal bladder tissues based on the TCGA dataset. *CNTD2*-related signaling pathways were identified through the GSEA. We analyzed the associations of *CNTD2* expression and bladder cancer progression and survival using GSE13507. Compared with 19 cases of normal bladder tissue, *CNTD2* gene expression was increased in 411 cases of bladder cancer. The high expression of *CNTD2* strongly correlated with grade (*P* < 0.0001), T classification (*P* = 0.0001), N classification (*P* = 0.00011), M classification (*P* = 0.044), age (*P* = 0.027), and gender (*P* = 0.0012). Bladder cancer patients with high *CNTD2* expression had shorter overall survival (*P* < 0.001). In the meantime, univariate and multivariate analyses showed that the increased expression of *CNTD2* was an independent factor for poor prognosis in bladder cancer patients (*P* < 0.001 and *P* < 0.001, respectively). *CNTD2* expression is closely related to bladder cancer progression, and the high expression of *CNTD2* may be an adverse biomarker in bladder cancer patients.

## Introduction

Bladder cancer is one of the most common genitourinary tract malignancies in the world (Torre et al., 2015) and the fourth most common carcinoma for males (Siegel et al., 2018). About 3.4 million people were affected by bladder cancer in the world, with 430,000 new cases each year since 2015 (Payton, 2013; Disease et al., 2016). The incidence and death rates of bladder cancer have also been rising rapidly in China in the past few decades (Pang et al., 2016; Liu et al., 2018). Despite the advancements in both local and systemic treatment, the progression and recurrence rates of non-invasive bladder cancer are still high, while survival rates for invasive and metastatic cancers are low (Jiang et al., 2019). Recurrence or metastasis of bladder cancer may refer to complex molecular regulatory mechanisms (Spiess et al., 2017; Wu et al., 2017). The biological characteristics and molecular regulatory mechanisms of bladder cancer have been studied extensively, and some progress has been made. However, the gene regulatory network of bladder cancer remains obscure (Jones et al., 2016; Yoshida et al., 2017). Therefore, it is urgent to study the pathogenesis and molecular regulation of bladder cancer. The exploration of suitable new therapeutic targets is a potential direction for the diagnosis and treatment of this disease.

The deregulation of cell cycle control is a common characteristic of cancer. Cyclin-dependent kinases (CDKs) were discovered in 1982, and were found to control the process of the cell cycle through interaction and activation of cyclins (Casimiro et al., 2012). CDKs are highly conserved serine/threonine kinases that must bind to cyclins to phosphorylate their substrates (Malumbres and Barbacid, 2009). Specific cyclin-CDK complexes are related to each important transition in the cell cycle. Many carcinomas exhibit inappropriate expression of typical cyclin cells. While most of the research conducted has focused on the function of canonical cyclins, the function of other proteins displaying the same characteristic “cyclin box,” which defines the CDK binding (Murray and Marks, 2001; Malumbres and Barbacid, 2005; Malumbres, 2014), remains largely unknown. This group of cyclins was called “atypical” due to their structural characteristics (Sánchez-Botet et al., 2018). *CNTD2*/*CCNP* is one of the atypical cyclins, and is known to overexpress in colon cancer and lung cancer. *CNTD2* may be a prognostic biomarker and therapeutic target in these two cancers (Gasa et al., 2017; Sánchez-Botet et al., 2018). However, the prognostic role of *CNTD2* in bladder cancer remains unclear.

Here, we investigated the relationship between *CNTD2* expression and bladder cancer. In the first place, we investigated the different expressions of *CNTD2* in 411 bladder cancers and 19 normal bladder tissues based on data obtained from The Cancer Genome Atlas (TCGA). Secondly, in order to detect the biological pathways involved in the *CNTD2* regulatory network related to the pathogenesis of bladder cancer, we conducted Gene Set Enrichment Analysis (GSEA). Finally, we surveyed the relationship between *CNTD2* expression and bladder cancer progression, stage, and survival through GSE13507. Our results suggest that high *CNTD2* expression is an adverse indicator of bladder cancer and is associated with poor outcomes.

## Materials and Methods

### Data Source

This study included 411 bladder cancers and 19 normal bladder tissues from the TCGA official website^1^. In order to investigate the role of *CNTD2* expression in the development of bladder cancer, we chose GSE13507 because GSE13507 has detailed clinical data information that can be used for subsequent analysis. GSE13507 was downloaded from the Gene Expression Omnibus (GEO) database^2^, which was published on May 6, 2010. In GSE13507, the researchers used 165 primary bladder cancer samples, 23 recurrent non-muscle invasive tumor tissues, 58 normal-looking bladder mucosae surrounding cancer, and 10 normal bladder mucosae for microarray analysis. The gene expression data of all patients were assessed by the Illumina Human-6 v2.0 Expression BeadChip. The design, quality control, and data normalization of all chip experiments conform to the standard Illumina protocol. The clinical data of GSE13507 was also downloaded from the NCBI GEO databases (see text footnote 2).

### Data Analysis

**FIGURE 1:**
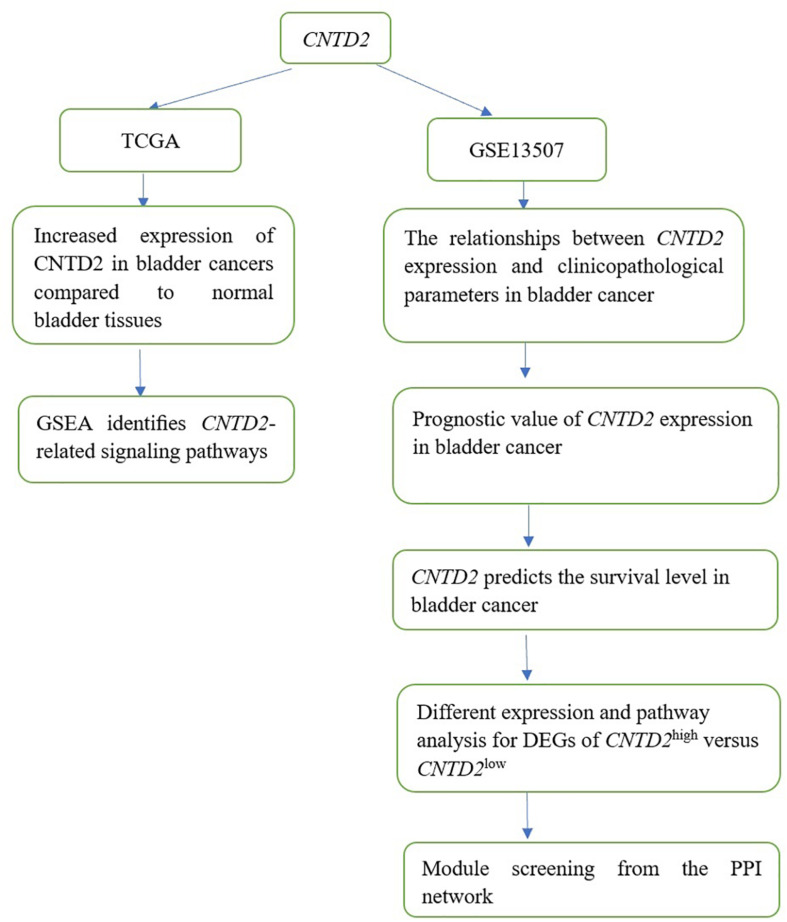
The flowchart of the research scheme of the article.

The different expressions of *CNTD2* between 411 bladder cancers and 19 normal bladder tissues from the TCGA were investigated. GSEA was used to identify *CNTD2*-related signaling pathways. GSE13507 was identified in GEO, patients with *CNTD2* expression values above the median were considered as *CNTD2*^high^, and the others were classified to be *CNTD2*^low^ in all patients with bladder cancer. *P*-value < 0.05 in unpaired *t* test analysis and fold change (FC, log2) > 0.5 or ≤0.5 were utilized to determine the differential expression of genes (DEGs) (Figure 1).

The experiment design, data normalization, and quality control conform to the standard scheme. The study was carried out in accordance with the International Conference and the Helsinki Declaration.

### Statistical Analysis

All statistical analysis was conducted by R software 3.6.2. The GSE13507 was divided into two groups (*CNTD2*^high^, *n* = 82; *CNTD2*^low^, *n* = 83) based on the median values of *CNTD2* expression. The limma package was used to test differential gene expression (Ritchie et al., 2015). The Cox regression multivariate analysis and the Kaplan-Meier method were used to evaluate the survival analysis, with group comparisons made using the log-rank test. Gene Ontology (GO) enrichment terms and Kyoto Encyclopedia of Genes and Genomes (KEGG) pathways were conducted through the clusterProfiler package (Yu et al., 2012).

## Results

### Increased Expression of *CNTD2* in Bladder Cancer Tissues

**FIGURE 2:**
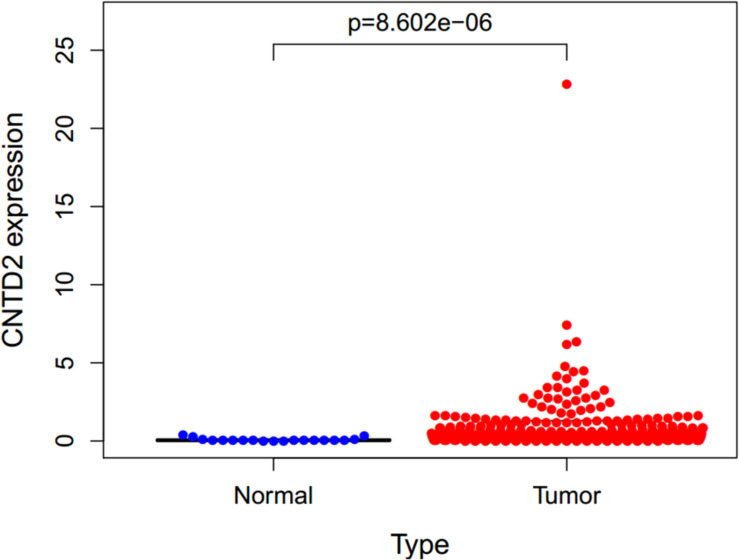
Expression of *CNTD2* in 411 bladder cancers and 19 normal bladder tissues. DNA replication, RNA polymerase, base excision repair, and homologous recombination.

The gene expressions data of 411 bladder cancers and 19 normal bladder tissues were downloaded from the TCGA. The *CNTD2* gene was highly expressed in bladder cancers compared with normal bladder tissues (Figure 2).

### GSEA Identifies *CNTD2*-Related Signaling Pathways

**FIGURE 3:**
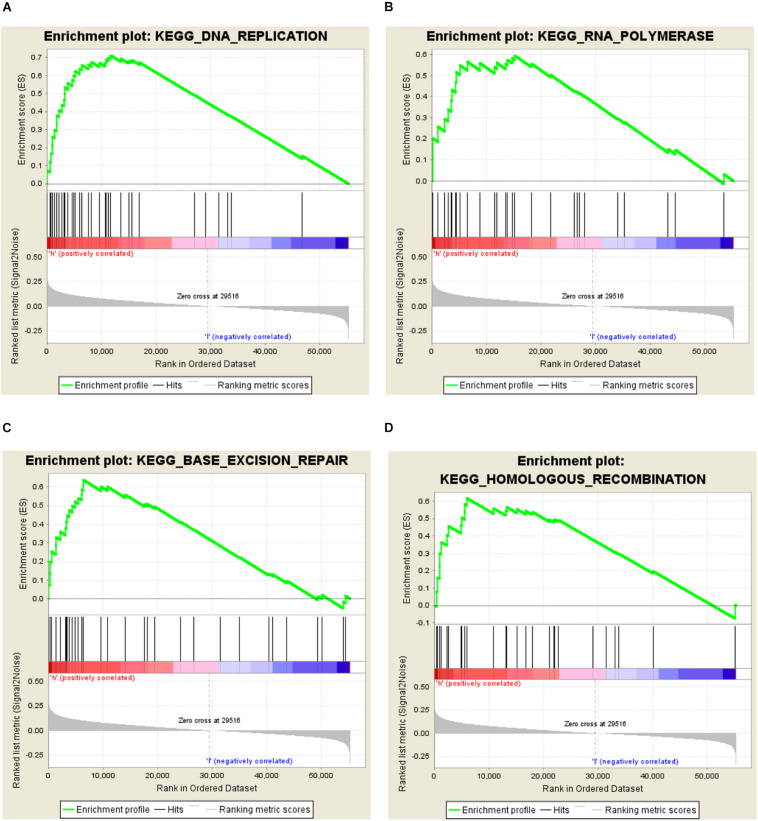
Enrichment plots from the Gene Set Enrichment Analysis (GSEA). GSEA results showing DNA replication **(A)**, RNA polymerase **(B)**, base excision repair **(C)**, and homologous recombination **(D)** are differentially enriched in the *CNTD2*^high^ expression phenotype.

In order to investigate the different activation signaling pathways in bladder cancer, we performed GSEA between high and low *CNTD2* expression datasets. GSEA was downloaded from https://www.gsea-msigdb.org/gsea/index.jsp. GSEA showed significant differences (NOM *P*-value < 0.05, FDR < 0.5) in the enrichment of the MSigDB collection (c2.cp.biocarta and h.all.v6.1.symbols). The most significant enrichment signaling pathways were selected according to the normalized enrichment score (NES) (Figure 3 and Table 1). Figure 3 demonstrates that DNA replication, RNA polymerase, base excision repair, and homologous recombination are differentially enriched in the *CNTD2*^high^ expression phenotype.

**TABLE 1:** Gene sets enriched in the high expression phenotype.

MSigDB collection	Gene set name	NES	NOM *P*-value	FDR *q*-value
c2.cp.biocarta.v6.1.symbols.gmt	KEGG_DNA_REPLICATION	1.684	0.049	0.233
h.all.v6.1.symbols.gm	KEGG_RNA_POLYMERASE	1.698	0.04	0.338
	KEGG_BASE_EXCISION_REPAIR	1.786	0.012	0.405
	KEGG_HOMOLOGOUS_RECOMBINATION	1.723	0.038	0.349

*Gene sets with NOM *P*-value < 0.05 and FDR *q*-value < 0.5 are considered as significant.*

*NES, normalized enrichment score; NOM, nominal; FDR, false discovery rate.*

### The Relationships Between *CNTD2* Expression and Clinicopathological Parameters in Bladder Cancer

**FIGURE 4:**
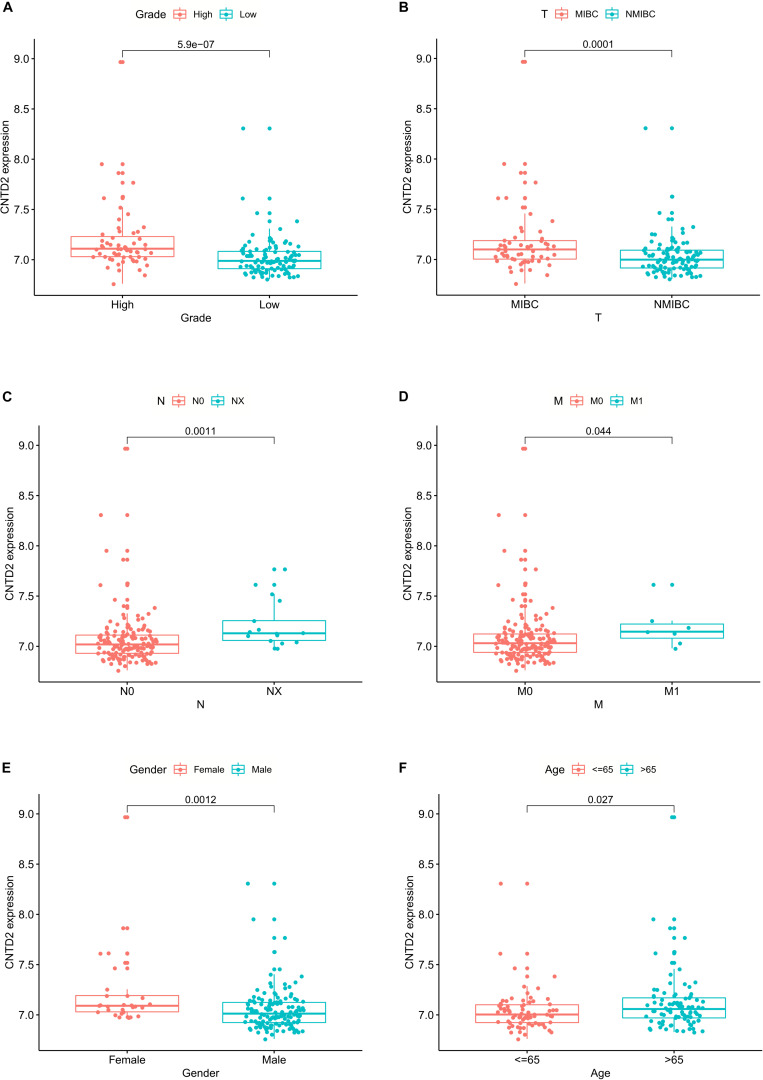
The relationships between *CNTD2* expression and clinicopathological parameters in bladder cancer.

We investigated the relationships between *CNTD2* and clinicopathological characteristics using the GSE13507 dataset of 165 bladder cancer patients. Fuhrman grade is closely related to the differentiation of bladder cancer and the prognosis of patients. TNM stage is a very important classification of bladder cancer. T represents tumor size, N represents lymph node metastasis, and M represents distant metastasis. As shown in Figure 4, the high expression of *CNTD2* strongly correlated with Fuhrman grade (*P* = 0.000), T classification (*P* = 0.000), N classification (*P* = 0.001), M classification (*P* = 0.044), gender (*P* = 0.001), and age (*P* = 0.027). The above results suggest that the high expression of *CNTD2* is correlated with the prognosis of bladder cancer.

### Prognostic Value of *CNTD2* Expression in Bladder Cancer

**FIGURE 5:**
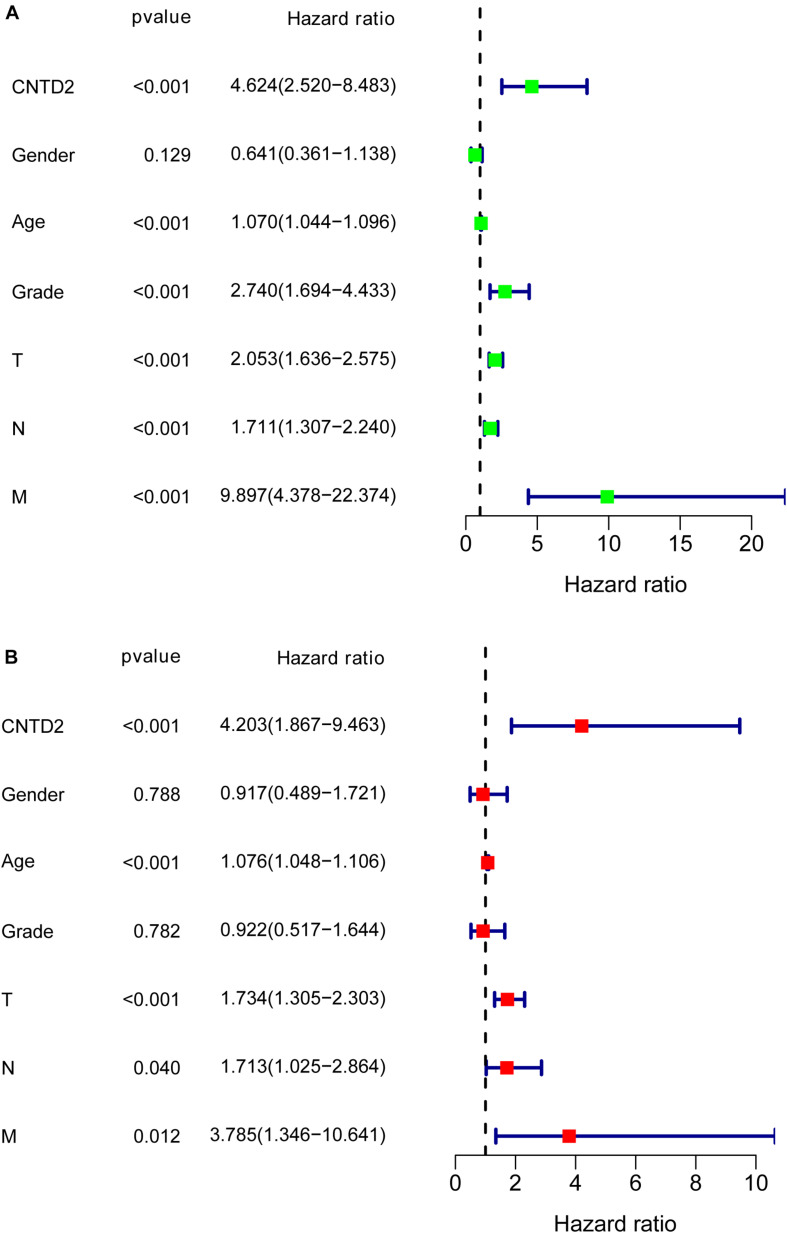
Cox regression analysis for the overall survival rates of bladder cancer patients.

We calculated multivariate hazard ratios for different variables using a Cox regression model in 165 patients with bladder cancer from the GSE13507 dataset. Univariate analysis results demonstrated that *CNTD2* overexpression (*P* < 0.001), age (*P* < 0.001), grade (*P* < 0.001), T classification (*P* < 0.001), N classification (*P* < 0.001), and M classification (*P* < 0.001) were all closely correlated with a poor prognosis (Figure 5A). Furthermore, multivariate analyses revealed that *CNTD2* overexpression (*P* < 0.001), age (*P* < 0.001), and T classification (*P* < 0.001) were all independent predictors of an unfavorable prognosis (Figure 5B). In summary, the results showed that overexpression of *CNTD2* could be used as a potential marker in the prognosis of patients with bladder cancer.

### *CNTD2* Predicts the Survival Level in Bladder Cancer

**FIGURE 6:**
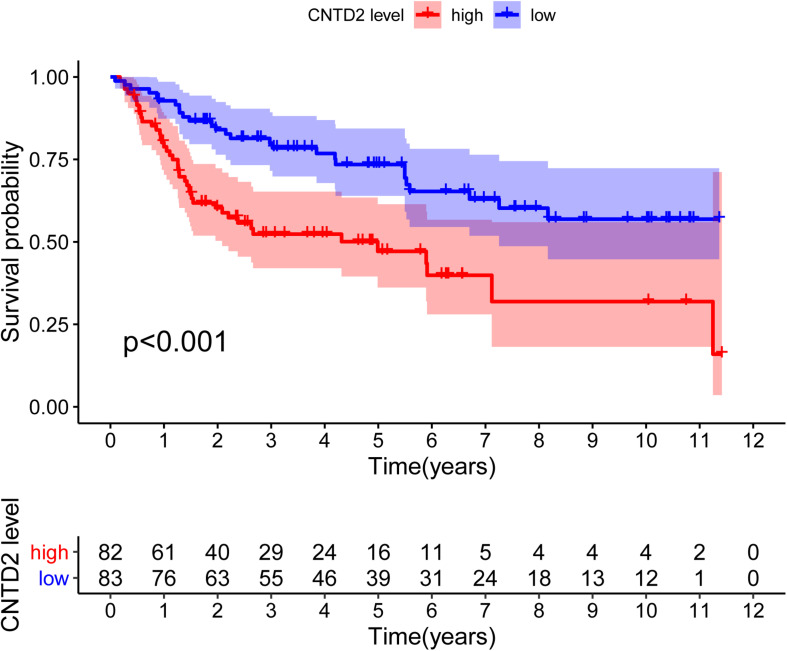
Overall survival curves of bladder cancer patients with high expression versus low expression of *CNTD2*.

According to the median expression of *CNTD2*, we separated the patients into two groups: *CNTD2*^low^ (*n* = 83) and *CNTD2*^high^ (*n* = 82). These results suggest that the high expression of *CNTD2* is related to the poor prognosis of bladder cancer. Thus, we further investigated the level of survival in the GSE13507 dataset. The results demonstrated that the *CNTD2*^high^ group had significantly shorter OS compared with the *CNTD2*^low^ group (Figure 6, *P* < 0.001).

### Different Expression and Pathway Analysis for DEGs of *CNTD2*^high^ Versus *CNTD2*^low^

**FIGURE 7:**
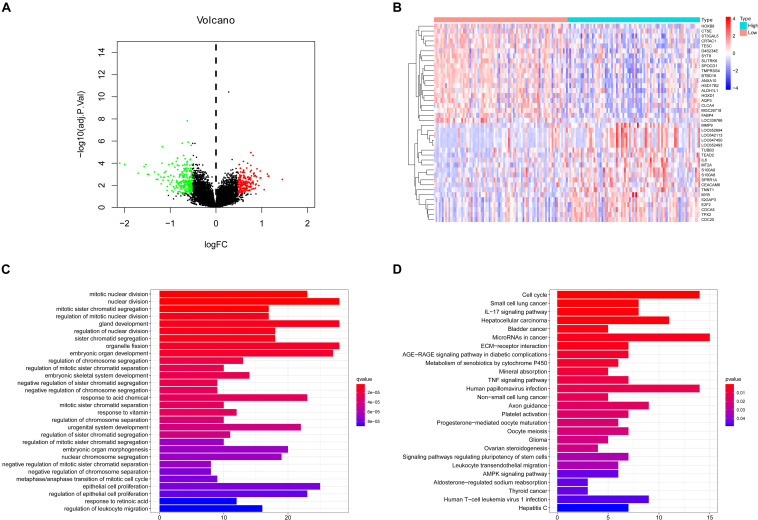
Different expression genes (DEGs), GO enrichment, and KEGG pathway analysis. **(A)** Volcano plot of the DEG expression between *CNTD2*^high^ and *CNTD2*^low^. Cutoff criteria for DEG significance was *P* < 0.05 and the absolute value of the log2 fold change > 0.5. The red dots represent 211 upregulated genes, the green dots represent 246 downregulated genes, and black dots denote non-significant genes. **(B)** Heatmap demonstrates the top 20 upregulated genes and the top 20 downregulated genes. Red indicates high expression, white indicates intermediate expression, and blue represents low expression. **(C,D)** GO and KEGG analysis show different enriched pathways for differential expression genes.

For the sake of finding the genes related to *CNTD2*, we compared the differential expression values of *CNTD2*^high^ versus *CNTD2*^low^. There were 246 downregulated genes and 211 upregulated genes [(FC, log2) > 0.5 or ≤0.5, *P* < 0.05, Figure 7A]. The first 20 downregulated and 20 upregulated genes were revealed in the heatmap (Figure 7B). We investigated the enriched GO terms and KEGG pathways using DEGs. In the biological process of GO, most DEGs are enriched in mitotic nuclear division (GO:0140014), nuclear division (GO:0000280), mitotic sister chromatid segregation (GO:0000070), regulation of mitotic nuclear division (GO:0007088), gland development (GO:0048732), regulation of nuclear division (GO:0051783), sister chromatid segregation (GO:0000819), organelle fission (GO:0048285), and embryonic organ development (GO:0048568) (Figure 7C). In the KEGG analysis results, cell cycle (hsa04110), small cell lung cancer (hsa05222), IL-17 signaling pathway (hsa04657), hepatocellular carcinoma (hsa05225), bladder cancer (hsa05219), and microRNAs in cancer (hsa05206) were the most enriched pathways (Figure 7D).

### Module Screening From the Protein-Protein Interaction Network

**FIGURE 8:**
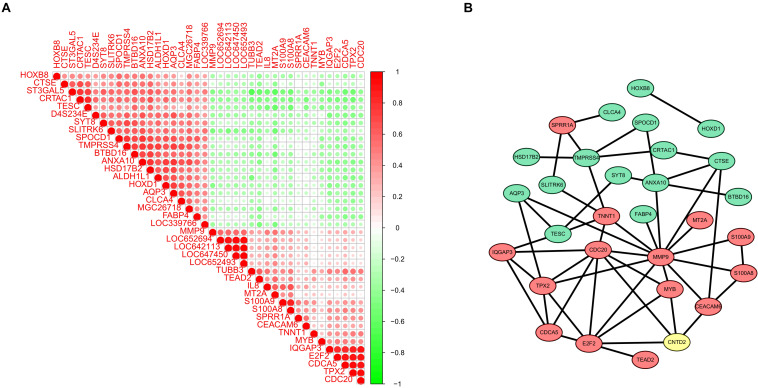
The correlation analysis and PPI results of DEGs. **(A)** The correlation analysis of DEGs through the Pearson correlation coefficient; the green dots mean negative correlation, while the red dots mean positive correlation. **(B)** PPI network of the top DEGs; green indicates downregulated genes, while red represents upregulated genes.

In the end, we calculated the correlativity between the top 40 DEGs of *CNTD2*^high^ versus *CNTD2*^low^ (Figure 8A). We also screened the protein–protein interaction (PPI) network in the STRING database using the top 40 DEGs and *CNTD2*. Most of the downregulated genes and the upregulated genes interact in the PPI network (Figure 8B), and *CDC20*, *MYB*, *CEACAM6*, and *MMP9* were all reported to be associated with cancer.

## Discussion

Bladder cancer is one of the leading causes of cancer death in the world (Spiess et al., 2017). After the diagnosis of bladder cancer, most patients showed a significant decline in health-related quality of life scores over time (Smith et al., 2018). The patient often develops a new lesion with a more advanced stage when bladder cancer is discovered anywhere within the urothelial tract. Bladder cancer is one of the deadliest tumors in the world, but its intracellular mechanism is still unknown. Therefore, it is necessary to explore new oncogenes to help in the early diagnosis of bladder cancer and predict the prognosis of patients with bladder cancer.

*CNTD2* is an atypical cyclin whose function is not well understood until now. It is currently speculated that *CNTD2* may be related to the cell cycle and even the development of cancers. *CNTD2* has been shown to be overexpressed in colon and lung cancers, and it may be a prognostic marker and therapeutic target in these two cancers (Gasa et al., 2017; Sánchez-Botet et al., 2018). However, the prognostic role of *CNTD2* in bladder cancer remains unclear.

In order to investigate the relationship between *CNTD2* and bladder cancer, we performed this research. Our results exhibited that the *CNTD2* gene is highly expressed in bladder cancers compared with normal bladder tissues. The *CNTD2*^high^ expression phenotype was associated with DNA replication, RNA polymerase, base excision repair, and homologous recombination by GSEA. Importantly, some studies have shown that DNA replication (Preston et al., 2010; Suzuki and Takahashi, 2013; Mughal et al., 2019), RNA polymerase (Marshall and White, 2008; Ferreira et al., 2020), base excision repair (Wallace et al., 2012; Marsden et al., 2017), and homologous recombination (Bishop and Schiestl, 2001) are all associated with the development of cancers.

In our research, we also found that the high level of *CNTD2* expression was related to an upward trend in bladder cancer progression. The low expression of *CNTD2* in bladder cancer patients was pertinent to favorable prognosis. Our results showed significant correlations between *CNTD2* expression and Fuhrman grade, T classification, N classification, M classification, gender, and age, which suggest that *CNTD2* expression might be important for the acquisition of malignant potential in bladder cancer. Furthermore, multivariate Cox regression analysis showed that *CNTD2* expression was an independent prognostic factor affecting overall survival in bladder cancer patients. Thus, the results of our study suggested that *CNTD2* could be a valuable biomarker for the prediction of bladder cancer prognosis, and to the best of our knowledge, this is the first report to address that *CNTD2* is associated with poor prognosis in bladder cancer.

The GO analysis showed that most DEGs were enriched in mitotic nuclear division, nuclear division, mitotic sister chromatid segregation, regulation of mitotic nuclear division, gland development, regulation of nuclear division, sister chromatid segregation, organelle fission, and embryonic organ development. The KEGG pathways were mainly enriched in cell cycle, small cell lung cancer, IL-17 signaling pathway, hepatocellular carcinoma, bladder cancer, and microRNAs in cancer. All of these results show that *CNTD2* may be a tumor oncogene in bladder cancer.

We found that many cancer-related genes interact with *CNTD2* through the PPI network. Gayyed et al. (2016) reported that high expression of *CDC20* was closely related with advanced tumor stage in carcinoma of the colon, breast, endometrium, and prostate. Choi et al. (2013) demonstrated that elevated *CDC20* expression is associated with poor prognosis in bladder cancer. *CEACAM6* is a member of the CEA family. Abnormal overexpression of *CEACAM6* plays a role in several characteristics of cancer, including uncontrolled proliferation, anoikis resistance, neovascularization, immune avoidance, invasion, and metastasis (Johnson and Mahadevan, 2015; Lee et al., 2015; Rizeq et al., 2018). *MYB* has now been investigated as an oncogene that is involved in some human leukemias. In addition, recent research suggests that *MYB* is activated in colon and breast cancers (Ramsay and Gonda, 2008).

In summary, the expression of *CNTD2* may be a potential prognostic molecular marker for predicting the survival of bladder cancer. Combined with the above GO and KEGG analysis results, *CNTD2* might interact with other bladder cancer-associated genes and was mainly involved in mitotic nuclear division, nuclear division, mitotic sister chromatid segregation, regulation of mitotic nuclear division, gland development, regulation of nuclear division, sister chromatid segregation, organelle fission, and embryonic organ development. Further experimental validation should be performed to prove the biologic impact of *CNTD2*. Nevertheless, our study is mainly based on the GSE13507 dataset, and further studies are still needed for verification.

## Data Availability Statement

The original contributions presented in the study are included in the article/supplementary material, further inquiries can be directed to the corresponding author/s.

## Ethics Statement

The studies involving human participants were reviewed and approved by Ethics Committee of Zhongshan People’s Hospital. The patients/participants provided their written informed consent to participate in this study. Written informed consent was obtained from the individual(s) for the publication of any potentially identifiable images or data included in this article.

## Author Contributions

WD and RY conceived and designed the study. MG data collected and performed the analyses. MG, ES, and GH drafted the manuscript. MG, WN, and WD prepared the figures and table. All authors reviewed and revised the manuscript.

## Conflict of Interest

The authors declare that the research was conducted in the absence of any commercial or financial relationships that could be construed as a potential conflict of interest.

## Footnotes

1 https://cancergenome.nih.gov/

2 https://www.ncbi.nlm.nih.gov/geo
